# Regular Meeting Fulton County Medical Society, October 16, 1913

**Published:** 1913-11

**Authors:** R. R. Daly


### REGULAR MEETING FULTON COUNTY MEDICAL SOCIETY.

October 16, 1913.

Reported by R. R. Daly, M. D.

Dr. W. S. Goldsmith reported several cases of operations upon the kidney and bladder for stone. These were illustrated by skiagraphs that were taken as aids to diagnosis.

A child five years old, had recurrent stone in the bladder. At a previous operation, sutures had been used in such a way that a thread remained exposed in the bladder and around this as a nucleous there had grown up two stones connected by the thread.

Another case in a child two years and seven months old, showed a small recurrent stone in the bladder. This is very unusual in one so young.

A third case was that in which there had been a bullet wound of the bladder some eight or nine years ago. The cystoscopic examination was impossible because of the irritation of the parts at the time relief was sought, but the X-ray showed the stone around the bullet and it was removed successfully.

Two kidney stones were exhibited that had come from a case of pyo-nephrosis and were found in the middle of the kidney. Another somewhat similar case was that of a lady in whom the kidney had been drained last July with relief of symptoms. It was found that the kidney was not functionating and it was removed. The stone was well within it. Skiagraphy had been objected to by the patient.

A large congenital cystic kidney was shown that had just been removed from a man of 26. He had suffered a slight injury a short time before the operation, and showed some signs of pyo-nephrosis. An operation for drainage was begun, but the kidney was removed as soon as the condition was exposed. The organ weighed 3 pounds, 10 ounces. The blow that caused him to come for relief simply accentuated a condition already present. He is doing well.

A case of tumor of the liver was described. It was thought to be a fibroma and was removed with the actual cautery. It proved later to be a gumma.

As the method of examination in children suspected of stone in the bladder, Goldsmith thought the X-ray was preferrable to the cystoscope.

Dr. E. G. Ballenger said that in the use of distilled drinking water, we had a good prophylactic to the formation of stones in the bladder. Harrison and Ochsner had both written on this subject.

Dr. J. L. Campbell said that he had seen the bullet case that Goldsmith reported over a year ago and that the diagnosis had been made at that time but that the man had left without treatment.

Dr W. B. Emory said that he has two cases of stone in children in which the diagnosis has been made without the X-ray. He agreed with Goldsmith that when there were symptoms of cystitis indicating stone in the least degree, the search should be thorough and repeated several times if necessary, for stone is more often present than it is credited with being. He has used lithia water to prevent recurrence without success.

Dr. E. C. Cartlege said that he understood that if the specific gravity were kept below 1015 by the drinking of any kind of water, stones would not form. The urine must be kept acid. Lithia tablets were contra-indicated because they contain acid to make them effervescing and this would tend to alkanalize the urine.

Dr. A. L. Fowler expressed the opinion that the cystoscope furnished the best means of diagnosing stone, even in children and that he used it in contradistinction to Dr. Goldsmith's experience.

Dr. Archibald Smith said that the chemic principals involved in the formation of stone should be considered when administering any remedy, water included. The uric acid stone having a decidedly different reaction from the phosphatic deposits.

Dr. Goldsmith said in closing that he would not attempt to discount the cystoscope in children's cases, but that as a rule it required an anaesthetic and took up a good deal of time. The X-ray could be applied to a two year old child in an instant and his experience with the skiagraphs has been satisfactory. He believes that all cases of enuresis should be skiagraphed in the search for the cause of the trouble.

The regular program was taken up as follows: "Chronic Prostatitis" by Dr. M. L. Boyd. "Surgical Kidney" by Dr. A. L. Fowler. "The Elimination of Gastric Disturbance from Oil of Sandalwood, Sodium Carbonate, Sodium Salicylate, Potassium Iodid, etc." by Dr. E. G. Ballenger. The papers by Drs. Fowler and Ballenger appear elsewhere in the Journal.

Dr. Goldsmith said of Dr. Fowler's paper that in many cases of pyelitis the injection of collargol for the purpose of skiagraphy did a lavage and introduced an antiseptic that greatly benefited the case at once. It never does any harm thus to wash out chronic cases.

Dr. L. Sage Hardin said that in the observation of hypertrophied thyroids at puberty there was a great deal of nervousness and Dr. Boyd's paper suggested that there might be some connection between the thyroid condition and the change in the development of the prostate at that time.

Dr. S. R. Roberts noted that Dr. Boyd had used the phrase "focus of infection" as applied to the prostate and ultimately causing septic arthritis. Taking, then, the prostate as the chief focus of infection in genito urinary cases, we have in abdominal cases the appendix, the gall bladder and the ovaries, while in nose and throat cases we have the tonsils, the accessory sinuses, etc. as primary foci of infection for arthritis. Arthritis is dus to a focus of infection somewhere and we must find this focus in order to be successful in treating all cases of arthritides.

The relation between renal calculus and pyelitis seemed to be jumped by Fowler as well as by Osler. Fowler said that there was some abrasion that would permit the action of germs to begin. Can calculus by itself produce constitutional symptoms? Is not a pyelitis indicated? He believes that there is such a thing as primary neuralgia of the kidney. He had two cases operated upon for the severe pains that might indicate some inflammation or presence of stone only to learn that there were no pathologic lesions in the kidneys.

As to Ballenger's paper, he would suggest the use of sodium iodide in the place of the potassium salt. It does not taste as bad and there are practically no gastric symptoms.

Dr. Hansell Crenshaw called attention to the relation between chronic prostatitis and the fatigue neuroses. Such a neurosis due to sexual processes will likely cause congestion of the prostate.

Dr. E. G. Ballenger said of Dr. Boyd's paper that he disagreed that there could be inflammation of the prostate without organisms. There may be considerable congestion, but wherever there was pus, he has been able to demonstrate micro organisms. The darkfield illumination will often show these better than any other method.

Dr. A. L. Fowler said of Boyd's paper that the fact that 40% of the cases had colon bacillus, goes to show that one can have chronic prostatitis who never has had gonorrhoea. In the Federal prison there are many mountaineers with prostatitis who never have been exposed to gonorrhoea. The reflex morbid neuroses in connection with this disease are important, Lassitude, referred pains, nervousness and various simulated conditions may thus originate.

Nothing in literature shows the presence of the gonococcus after the patient has had the disease for two years.

Pyelitis is not so much due to the stone in the kidney as to the crystals in the urine.

The treatment by means of the albumenates of silver is often efficatious. This, in connection with the Trendelenberg position has cured cases several times. Froid's suggestion of sexual neuroses in connection with the prostatitis is important, but in his opinion it is more often psychic than direct. The majority of neuroses in men is associated with prostatitis and posterior urethritis.

It is impossible to get cultures from the prostate alone. The urethra has an enormous number of organisms and these can not by any known method be completely eliminated in securing samples of the prostatic secretion.

Drf. J. E. Paullin said that several years ago he made about 80 cultures of the secretions of the prostate as well as he could get them. Seven or eight organisms were always present of which the colon bacillus was the prominent one. He was never able to show the gonococcus in the long standing cases.

---

### REGULAR MEETING FULTON COUNTY MEDICAL SOCIETY.

November 6, 1913.

Dr. J. S. Derr introduced a patient to the Society who gave the following account of himself. For many years he had been a sufferer with chronic constipation. He took all kinds of medicine with varying effects as to securing evacuation but always with the result that after the movement, it was of no further use. Some ten or more years ago he went twenty-eight days without a movement from the bowels and all during that time he was eating and drinking heartily. He was at a rifle practice meet where he made excellent score, during part of this time.

He had many physicians examine him without getting benefit until Dr. Derr had applied the X-ray, discovered the trouble and had him operated upon. Dr. Derr said that the colon had been injected and an ernormously dilated caecum was shown in the radiograph.

Dr. L. Sage Hardin had operated, resecting the caecum and all the dilated part of the gut. The recovery was quick and satisfactory. The patient has been well and normal since then.

Dr. F. W. McRae said that he had seen the patient but without the X-ray was unable to determine the cause. The operation certainly was the right one to perform and the only procedure that would cure.

Dr. F. W. McRae reported a case of nephrectomy in a lady who for eighteen years had been a profound sufferer with frequent attacks of renal colic. The pains had been so numerous that she became an habitual user of morphine. The skiagraph of Dr. Derr showed stones in the kidney. The sulphopthalein test had showed return of 6½% in the diseased side and but 18% in the other. Neither kidney was doing much but it was clear that the diseased one was of no use.

The kidney was removed to-day and found to be a mass as large as a six months child but almost entirely cystic. There were several stones as located in the skiagraph and these together with the cystic kidney were shown to the Society.

Dr. F . K. Boland read a paper on Acute Osteo-myelitis.

Dr. F. W. McRae said that he had done no bone surgery recently and was not in immediate touch with all that was being done in that line. In one of these cases however, he had a most unpleasant association. About ten years ago he was sued for $10,000 damages. Twenty years ago, he saw a case of acute osteomyelitis several days after the trauma. The joint was then swollen badly and it looked like rheumatism. The treatment lasted a year without benefit and then the case passed to other hands. He felt that he had done all he could owing to the extent of progress it had made when brought to him. Ten years later, the limb had to be amputated for chronic osteomyelitis and then it was that he was sued. Since then whenever he has come into contact with a case he has been sure that early operation was advised and the responsibility placed where it belonged.

He had another case of a little girl who died in two weeks from metastatic abscesses.

The diagnosis should be easy according to Dr. Boland's paper. The age of the patient from childhood to twenty-five, the history of trauma or exposure or the antecedent depressing fevers; the peculiar deep boring pain with difficulty in the child's locating it but at the same time an acutely sensitive spot when the surgeon found the focus, all these, aided when possible by the X-ray should tell the story accurately. The indication for prompt operation is as urgent as in appendicitis; even a few hours making a great difference in the extent of the bone involved and the amount of work needed.

Dr. E. L. Griffin said that the discussion of osteomyletis is timely and should be extended to all the profession by the paper.

Rheumatism is very often given as a mistaken diagnosis and this should be corrected. He, himself, doubts that he has ever seen articular rheumatism in a young child. One should always be suspecting osteomyelitis when there is pain in a long bone and particularly at the epiphises. The disease may be slow, especially in tubercular cases. The white swellings frequently reported as such are often enough osteomyelitis. The infection may come from the exanthemata and from the tonsils, in his opinion as well as from a direct trauma.

The treatment is to open at once, and no matter how small the area, it should be thoroughly cleansed and the medullary canal opened to be sure there was no disease within. Then it is most important that the denuded bone surface should be so broad and flat that the periosteum should have ample opportunity to rest down upon it and resume its function. A narrow groove will not permit this and a secondary operation will become necessary.

Dr. E. C. Thrash said that there are three reasons why osteomyelitis is a child's disease.

1. The young tissues are not highly resistant to the growth of bacteria.

2. Bacteria pass more readily through the lung and enter the circulation by the lymphatics in the young. They pass into the arterioles at the injured part—and here in the bones the circulation is terminal—and thus set up the inflammation. In later life the lung acts as a bulwark against this method of invasion.

3. In adult life we establish an immunity to this special disease by our absorption of so many other bacteria.

Dr. E. G. Jones expressed his appreciation of a paper on this subject. The cases that have come to him have all been called rheumatism in advance, showing the prevalence of that idea. In all his cases hereafter he will enter the medullary canal whether pus shows under the periosteum or not. He has had once to reopen a case because of the pus in the canal when at the first operation there was no sign of it.

Dr. B. S. Moore said he thought that one should not invariably think of the long bones in regard to this disease. The crest of the illium has been involved in one of his cases. Deep pains in the back might indicate the same thing.

Dr. J. S. Derr said that his experience had not brought such cases for X-ray examination. He thought that pus would show under the periosteum, but asked if anyone knew of its showing in the marrow.

Dr. Boland closed by saying that the disease began most frequently in the marrow and that the X-ray had shown it there.

Dr. J. S. Derr read a paper on the "X-ray Examination of the Gastro-Intestinal Tract." It was profusely illustrated with skiagraphs. The illuminated plates clearly showed all the points covered. The paper will appear elsewhere in the Journal.

Dr. Frank Byrd said that his personal experience in skiagraphy has been in bones and he realizes that only the utmost skill in technic coupled with a profound study on the parts shown can be successful in interpreting Gastro-Intestinal work. He commended the paper highly.

Dr. Michael Hoke said that it was easy to take pictures, but it required an expert to know just where and under what conditions to make the exposures and after that, expert knowledge was demanded to interpret them. Dr. Derr was thus equipped and he should be consulted in the first instance instead of waiting to see the results of less skilled trials.

Drs. Michael Hoke and F. G. Hodgson presented "Lantern Slides and Moving Picture Exhibitions of Orthopaedic Cases."

There was no set paper, but as the pictures were shown, explanations and interpretations were given. A child was shown on the screen so that the deformity of the club feet could be seen in the distance. Then a closer picture would portray front, back and side views of the deformity just at the joint itself. Thus one gained a comprehensive idea of the ordinary external appearance of the limb and its peculiar twist. Next appeared on the screen the various skiagraphs showing the arrangements of the bones within the joint and along the leg. The mechanical problem of reshaping the bones of the metatarsus, tarsus and leg could be indicated and was readily comprehended as the pointer marked the shadows while the operator explained them. Weeks later after the various operations had been performed and there was recovery enough to discharge the child from the surgery, other photographs showed the reformed condition and skiagraphs revealed the internal arrangements according to the plans the operators had previously indicated. Thus club feet of all forms were repaired and useful ankles were demonstrated. Other feet were moved backward or forward so as to replace them under the lines of weight and enable the patient to balance with ease. A broken arm with lack of union was manipulated with splint and pressure until it was firm and useful. The method being so clearly shown as to require almost no explanation. A fibula was transplanted in the place of a tibia that had been removed in one third of its length in a case of chronic osteomyelitis. In connection with Dr. Boland's paper it was very interesting to see that the upper portion of the tibia that remained in place after the operation for chronic osteomyelitis still contained the dormant disease and soon after the transplanting operation, it livened up and had to be removed. This had been foreseen as to the possibility at least, and the fibula had not been attached to it above.

But not only were these pictures showing immediate results, displayed. In case after case five years had elapsed before the next set of views were taken. Here the cinematograph was called in and moving pictures of cases, five years after operations had been performed in this radical way were shown. The patients well, moving about easily and comfortably.

They walked with shoes and barefoot before the cameras so that one could see every motion. The joints were evidently serviceable and aside from the scars there was no deformity. The muscles had developed and the parts generally had grown in proportion to the rest of the body, almost as well as the perfect child.

One specially interesting case was that of the transplanting of the peronei in case of anterior tibial paralysis. It was successful.

